# Enhanced Expression of Alcohol Dehydrogenase I in *Pichia pastoris* Reduces the Content of Acetaldehyde in Wines

**DOI:** 10.3390/microorganisms12010038

**Published:** 2023-12-25

**Authors:** Kun Geng, Ying Lin, Xueyun Zheng, Cheng Li, Shuting Chen, He Ling, Jun Yang, Xiangyu Zhu, Shuli Liang

**Affiliations:** 1 School of Biology and Biological Engineering, South China University of Technology, Guangzhou 510006, China; 2 Guangdong Provincial Key Laboratory of Fermentation and Enzyme Engineering, South China University of Technology, Guangzhou 510006, China; 3 Key Laboratory of Fermentation Engineering of Ministry of Education, School of Biological Engineering and Food, Hubei University of Technology, Wuhan 430068, China; 4 Department of Biology, Massachusetts Institute of Technology, Cambridge, MA 02139, USA

**Keywords:** alcohol dehydrogenase I, *Pichia pastoris*, cofactor regeneration, multicopy, acetaldehyde, wine

## Abstract

Acetaldehyde is an important carbonyl compound commonly detected in wines. A high concentration of acetaldehyde can affect the flavor of wines and result in adverse effects on human health. Alcohol dehydrogenase I (ADH1) in *Saccharomyces cerevisiae* catalyzes the reduction reaction of acetaldehyde into ethanol in the presence of cofactors, showing the potential to reduce the content of acetaldehyde in wines. In this study, *ADH1* was successfully expressed in *Pichia pastoris* GS115 based on codon optimization. Then, the expression level of *ADH1* was enhanced by replacing its promoter with optimized promoters and increasing the copy number of the expression cassette, with ADH1 being purified using nickel column affinity chromatography. The enzymatic activity of purified ADH1 reached 605.44 ± 44.30 U/mg. The results of the effect of ADH1 on the content of acetaldehyde in wine revealed that the acetaldehyde content of wine samples was reduced from 168.05 ± 0.55 to 113.17 ± 6.08 mg/L with the addition of 5 mM NADH and the catalysis of ADH1, and from 135.53 ± 4.08 to 52.89 ± 2.20 mg/L through cofactor regeneration. Our study provides a novel approach to reducing the content of acetaldehyde in wines through enzymatic catalysis.

## 1. Introduction

Acetaldehyde is an important carbonyl compound commonly detected in wines, accounting for more than 90% of total aldehydes [1]. It is mainly produced through microbial fermentation as part of the brewing process; in particular, acetaldehyde can be produced either from the conversion of pyruvate catalyzed by pyruvate decarboxylase [2], or during wine aging. Indeed, copper or ferrous ions can catalyze the oxidation reaction of many substances in wines [3,4], including the oxidation reaction of ethanol to acetaldehyde.

Acetaldehyde can affect many aspects of wine quality, such as wine aroma, color, and texture. A low concentration of acetaldehyde enhances the pleasant fruity aroma in wines [1], while a high concentration of acetaldehyde causes an odor of either ripe apples or fresh grasses [5,6]. Furthermore, acetaldehyde can promote the polymerization of anthocyanins, catechin, and tannins in wines, ultimately improving the color stability and reducing the astringency in wines [1,6]. Moreover, acetaldehyde can also react with various types of substances such as flavonoids in wines to improve the color stability of wines [7]. Therefore, numerous studies have explored the quality improvement of wines by adding exogenous acetaldehyde [8,9]. However, acetaldehyde in wines is commonly considered “carcinogenic to humans” (IARC Group 1) [10] and is harmful to human health, increasing the risk of various types of cancer, such as oral cancer [11]. Therefore, it is necessary and important to reduce the content of acetaldehyde in wines in order to maintain the wine flavor and human health. At present, the regulation of acetaldehyde content in wine is achieved mainly through the optimization of the fermentation conditions of the brewing process, such as temperature and ventilation (Table 1).

Alcohol dehydrogenases (ADHs; E.C. 1.1.1.1) are oxidoreductases widely distributed in both eukaryotes and prokaryotes that play a critical role in alcohol and aldehyde metabolism [16]. ADHs can catalyze the reversible oxidation reaction of alcohols into either aldehydes or ketones in the presence of cofactors [17]. The substrates of ADHs mainly include primary unbranched aliphatic alcohols [18], such as ethanol. Many isoenzymes of ADHs are identified in *Saccharomyces cerevisiae*, with ADH1, one of the key enzymes in the ethanol metabolism of *S. cerevisiae*, catalyzing the conversion reaction of acetaldehyde into ethanol. ADH1 is a homotetramer enzyme of 150 kDa with a subunit of 37 kDa [19], which contains two zinc ions. One of these two zinc ions is located at the active site of the enzyme to play a key role in the catalytic process [17,20], while the other zinc ion maintains the structure of ADH1 [17]. Due to its capability of catalyzing the conversion of acetaldehyde into ethanol, ADH1 has shown significant potential in the wine industry to reduce the content of acetaldehyde in wines.

*Pichia pastoris*, commonly known as *Komagataella phaffii* and a Generally Recognized as Safe (GRAS) microorganism, is widely used in the production of industrial enzymes and pharmaceutical proteins [21]. As a methylotrophic yeast, *P. pastoris* can use a strong methanol-induced promoter, P*_AOX1_*, to achieve the high-level expression of recombinant proteins [22]. However, P*_AOX1_* is strictly repressed by common carbon sources, such as glycerol and glucose [21]. Therefore, the expression process of recombinant proteins driven by P*_AOX1_* can be divided into the cellular growth stage (i.e., glycerol or glucose is chosen as the carbon source) and the product generation stage (i.e., methanol is chosen as the carbon source), with the latter being conducive to promoting the production of recombinant proteins. Moreover, the high-density fermentation technology of *P. pastoris* has been gradually established, making it appropriate for industrial production. In the production of type III human-like collagens, the cell wet weight of *P. pastoris* could reach 270 g/L after 66 h high-density fermentation in a BSM medium [23]. Furthermore, as a eukaryotic expression system, *P. pastoris* contains many post-translational modifications, like those of higher eukaryotes, i.e., the correction of protein folding, the formation of disulfide bonds, and glycosylation modifications [21].

In this study, *ADH1* was expressed in *P. pastoris* GS115 with ADH1 purified using the nickel column affinity chromatography. Then, the enzymatic activities of ADH1_N-6×His_ (i.e., 6×His-tag was added to the N terminal of ADH1) and ADH1_C-6×His_ (i.e., 6×His-tag was added to the C terminal of ADH1) were comparatively evaluated. The expression level of ADH1 in *P. pastoris* GS115 was enhanced by optimizing the promoter and increasing the gene dosage (i.e., the copy number of the target gene). Finally, the effect of ADH1 on reducing the content of acetaldehyde in wine was evaluated. Overall, purified ADH1 with the addition of NADH could successfully reduce the content of acetaldehyde in wine, and the effect of ADH1 on reducing the content of acetaldehyde in wines could be further synergistically improved with the addition of GDH. In summary, this study provided a novel approach to reduce the content of acetaldehyde in wine through enzymatic catalysis.

## 2. Materials and Methods

### 2.1. Fungal and Bacterial Strains, Plasmids, Reagents, and Medium

*Escherichia coli* Top10 (Invitrogen, Waltham, MA, USA) was used as the host strain for plasmid clone. *Pichia pastoris* GS115 (Invitrogen, Waltham, MA, USA) was used for protein expression. Plasmids pPIC9K (Invitrogen, Waltham, MA, USA) and pHKA (Lab construction) [24] were used as expression vectors. The sequence of *ADH1* derived from *S. cerevisiae* (GenBank ID: KZV07794.1) was first subjected to codon optimization and then synthesized by Tsingke Biotechnology (Beijing, China). The codon-optimized sequence of ADH1 is provided in the Supplementary File “The codon-optimized sequence of ADH1 (5’→3’).” Restriction enzymes, T4 DNA ligase (Thermo Fisher Scientific, Waltham, MA, USA), and Seamless Assembly Cloning Kit (Clone Smarter, Beijing, China) were used for the construction of plasmids. The 2,4-dinitrophenylhydrazine (DNPH) (Macklin Biochemical, Shanghai, China) was used for the determination of acetaldehyde using precolumn derivatization. The acetaldehyde standard (Sigma-Aldrich, Steinheim, Germany) was used to fit the standard curve of acetaldehyde concentration. The β-NADH standard (Macklin Biochemical, Shanghai, China), glucose dehydrogenase (GDH) (Aladdin Biochemical, Shanghai, China), wine samples (Domaine Durieu, Cotes du Rhone Villages, France), and baijiu samples (Jiangji Winery, Chongqing, China) were used for the experiment of reducing the content of acetaldehyde in wine and baijiu.

NaCl, glucose, glycerol, methanol, ethanol (Damao Chemical, Tianjin, China), yeast extract, tryptone, agar, peptone (OXOID, Basingstoke, UK), and yeast nitrogen base (Sangon Biotech, Shanghai, China) were used in the preparation of medium. Kanamycin (Macklin Biochemical, Shanghai, China) was used for screening. An LB medium (1% NaCl, 0.5% yeast extract, and 1% tryptone; plate containing 2% agar) was used for the cultivation of *E. coli* Top10, and kanamycin with a final concentration of 50 ug/mL was added as necessary. MD medium (2% glucose, 1.34% yeast nitrogen base, and 2% agar) and BMGY/BMMY/BMEY [1.34% yeast nitrogen base, 1% yeast extract, 2% peptone, 100 mM phosphate-buffered saline (PBS) of pH 6.0, and 1% glycerol, 1% methanol, or 1% ethanol] were used for the cultivation of recombinant strains of *P. pastoris*.

### 2.2. Construction of Plasmids

To compare the enzymatic activities of the strains with expression of ADH1_N-6×His_ (with 6×His-tag added to the N terminal of ADH1) or ADH1_C-6×His_ (with 6×His-tag added to the C terminal of ADH1), plasmids pPIC9K-*ADH1_N-6×His_* and pPIC9K-*ADH1_C-6×His_* were constructed. Then, promoters P*_AOX1_*, P*_AOXm_* [25], and P*_ADH3_* [26] were used to induce the expression of ADH1, and plasmids pHKA-*ADH1_N-6×His_*, pHKAOXm-*ADH1_N-6×His_*, and pHKADH3-*ADH1_N-6×His_* were constructed. In order to further increase the expression level of ADH1, multicopy plasmids, i.e., pHKA-*ADH1_N-6×His_*-2Copies, pHKAOXm-*ADH1_N-6×His_*-2Copies, and pHKAOXm-*ADH1_N-6×His_*-3Copies, were constructed. The terminators of all expression plasmids were AOX1 terminator (AOX1 TT) (Table 2). Construction methods of plasmids were shown in the Supplementary File “Construction of plasmids”.

### 2.3. Construction and Cultivation of Recombinant Strains of Pichia pastoris

The expression plasmids were digested with the restriction enzyme *Kpn*2I and then transformed into the competent cells of *P. pastoris* GS115. MD medium plates were used to screen positive transformants, with the transformed cells incubated at 30 °C for 3 d. Each positive transformant was identified through PCR using the upstream and downstream primers of *ADH1* (Supplementary Table S1). The positive transformants of multicopy strains were identified through PCR using primers Copy-F and Copy-R, which were located upstream and downstream of the expression cassette, and the length of the target segments amplified using PCR was used to identify the multicopy strains. The positive transformants were then cultured in flasks, each containing 10 mL BMGY medium at 30 °C and 250 rpm, and shaken for 20 h. The seed liquid was then transferred to 25 mL BMMY or BMEY medium with an initial optical density at 600 nm (OD_600_) of 1. Specifically, strains with methanol-induced promoters P*_AOX1_* or P*_AOXm_* were cultured in BMMY, and strains with the ethanol-induced promoter P*_ADH3_* were cultured in BMEY. Methanol or ethanol was added every 24 h at the radio of 1% (*v*/*v*) for a total of 72 h cultivation.

### 2.4. Extraction of Intracellular Proteins

The extraction of a small quantity of intracellular proteins was performed as previously reported [27]. Briefly, cells were collected after 72 h cultivation and washed three times with 100 mM PBS (pH = 7.5). Then, 1 mL of the washed cells (OD_600_ = 50) was added into a cell disruption tube containing 0.75 g of 0.5 mm glass beads. After 8 cycles of high-speed homogenization, each for 30 s, and incubation in an ice bath for 1 min, the raw extractions were collected through centrifugation. Finally, the enzymatic activities of the extracted intracellular proteins were measured as described in Section 2.5. This approach was used to compare the expression levels among different strains of *P. pastoris*.

The extraction of the large quantity of intracellular proteins was performed as follows: cells were washed according to the method described above and then 50 mM Tris-HCl buffer (pH = 8.0) was added at a ratio of 10 mL per 1 g wet weight. The disruption of cells was performed using a high-pressure homogenizer and then the intracellular proteins were obtained by collecting the supernatant after the centrifugation of the fragmentized liquid. Finally, the large quantity of intracellular proteins was separated to obtain purified ADH1 via nickel column affinity chromatography.

### 2.5. Detection of the Enzymatic Activity of ADH1

According to the maximum absorption peak of NADH at 340 nm, the enzymatic activity of ADH1 was quantified by measuring the absorbance variation in the reaction system at 340 nm [28]. A series of NADH standard solutions, i.e., NADH was dissolved in 50 mM Tris-HCl buffer (pH = 8.0) with concentrations of 0, 0.2, 0.3, 0.4, 0.45, 0.5, and 0.6 mM, respectively, were prepared, and the standard curve of OD_340_ based on NADH concentrations was generated.

The enzymatic reaction solution contained 95 mM PBS (pH = 7.5), 0.5 mM NADH, and 2 mM acetaldehyde. A 3 μL sample was mixed with 247 μL of the reaction solution. Then, 200 μL of the mixture was collected to measure the absorption at 340 nm using a microplate reader. The values of OD_340_ were recorded every 5 s for a total of 3 min prior to the completion of the reactions. The enzymatic activity was calculated based on the OD_340_ curve with the linear variations. One enzymatic activity unit (U) of ADH1 was defined as the quantity of enzymes required to react with 1 μmol of NADH per min.

### 2.6. Purification of ADH1

The crude enzyme solution obtained from the extraction of the large quantity of intracellular proteins was purified via nickel column affinity chromatography using buffer A (50 mM Tris, 150 mM NaCl, and 10% glycerin; pH = 8.0) and buffer B (50 mM Tris, 150 mM NaCl, 10% glycerin, and 300 mM imidazole; pH = 8.0). The crude enzyme solution was first filtered through the 0.22 μm filter membrane prior to the purification. The elution processes were performed with gradient buffer B at 5%, 10%, 20%, 50%, 80%, and 100%, respectively, and samples at the peak positions were collected.

### 2.7. Detection of the Content of Acetaldehyde in Wine

The concentrations of acetaldehyde in wine samples were determined via high-performance liquid chromatography (HPLC) as previously described [29]. Acetaldehyde was pre-treated with DNPH prior to the detection. Then, the levels of reaction products, i.e., phenylhydrazones, were measured. A series of standard acetaldehyde solutions with concentrations of 5, 25, 50, 100, 200, 250, and 400 mg/L, respectively, were prepared using the 12% ethanol solution. The 10 g/L DNPH solution was prepared using warming and ultrasonic treatment [29].

The methods used for sample pretreatment were as follows: 0.5 mL sample, 0.5 mL acetonitrile, and 150 μL sulfuric acid (25%; *v*/*v*) were thoroughly mixed and then a total of 200 μL DNPH (10 g/L) was added to the sample at 30 °C for 15 min. The final reaction solution was filtered through a 0.22 μm filter membrane for the next round of detection.

The reaction product, phenylhydrazone, was detected via HPLC. The C18 column (4.6 × 250 mm, 5 μm) was used for the separation processes. The reaction temperature was maintained at 30 °C and the UV signal detector was set at 365 nm. The flow rate was 1.0 mL/min with an injection volume of 10 μL. The gradient elution procedure was performed with a total elution time of 30 min. The mobile phase A was a sodium acetate solution (25 mM; pH = 4.5) and mobile phase B contained the acetonitrile. The initial mobile phase A gradient was 50%, and the gradient was gradually changed to 40% within 0–15 min, 50% within 15–20 min, and then maintained at 50% for 20–30 min.

### 2.8. Application of ADH1 in Reducing the Content of Acetaldehyde in Wine

The reduction of acetaldehyde in wine by ADH1 was carried out as follows: the wine sample and enzyme solution were mixed at a ratio of 1.7 mL wine sample per 200 μL purified enzyme solution, and then 100 μL NADH solution was added to prepare 2 mL reaction system. The concentrations of NADH in the experimental groups were set to 0, 0.5, 1, 2, 3, and 5 mM, respectively. In the control groups, the concentration of NADH was set to 5 mM with the enzyme solution replaced with 50 mM Tris-HCl buffer. Each reaction system was incubated at 25 °C and 500 r/min. The concentration of acetaldehyde was determined at 0.5, 1.5, and 4 h incubation, respectively.

In order to recycle cofactors in the reaction system, together with ADH1, glucose dehydrogenase (GDH) was introduced to catalyze the conversion of glucose to gluconic acid and regenerate NADH [30]. Since wine may contain a certain amount of glucose [31], NADH was regenerated through the catalysis reaction of GDH. The reaction systems of the reduction of acetaldehyde in wine by ADH1 and GDH are shown in Supplementary Table S2. ADH1 solution, which was obtained in Section 3.3. (below), and GDH (2 mg/mL) and glucose solutions (40 mM) were prepared for the reactions of 4 experimental groups. Group 1 contained only ADH1, and NADH, GDH, and glucose were successively added in groups 2 to 4, i.e., group 2 contained ADH1 + NADH, group 3 contained ADH1 + NADH + GDH, and group 4 contained ADH1 + NADH + GDH + glucose, respectively. The concentrations of NADH in groups 2 to 4 were set to 0.5 mM. Each reaction system was incubated at 25 °C and 500 r/min, and the concentration of acetaldehyde was determined in 16 h incubation.

### 2.9. Statistical Analysis

Each experimental measurement was repeated with three biological replicates. Data were expressed as the mean ± standard deviation (SD). The statistical significance was determined using Student’s *t* test based on *p* < 0.05 (**) and *p* < 0.01 (***), respectively.

## 3. Results

### 3.1. Construction of Expression Vectors

**Figure 1:**
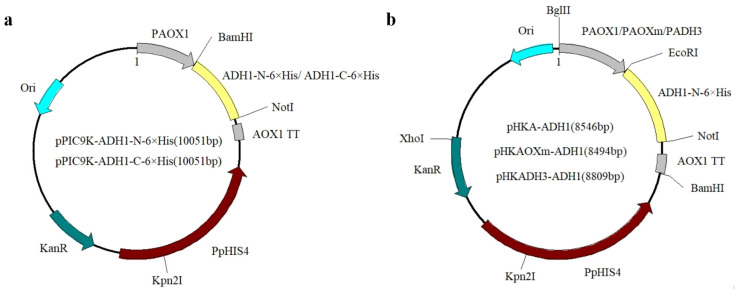
Diagram of the plasmids with a single expression cassette for the expression of *ADH1_N-6×His_* or *ADH1_C-6×His_* with promoter P*_AOX1_* (**a**) and for the expression of *ADH1_N-6×His_* with promoters P*_AOX1_*, P*_AOXm_*, or P*_ADH3_* (**b**).

The plasmids successfully constructed in this study are shown in Table 2 and the diagrams of single expression cassette plasmids are given in Figure 1a,b. The plasmids pPIC9K-*ADH1_N-6×His_* and pPIC9K-*ADH1_C-6×His_* were constructed through the digestion of restriction enzymes and T4 ligation. The fragments *ADH1_N-6×His_* and *ADH1_C-6×His_* were amplified by PCR with *Bam*HI and *Not*I restriction enzyme sites at both ends, and these two fragments and plasmid pPIC9K were digested with restriction enzymes *Bam*HI and *Not*I. Finally, the digested segments were ligated to obtain recombinant plasmids pPIC9K-*ADH1_N-6×His_* and pPIC9K-*ADH1_C-6×His_*. The plasmids pHKA-*ADH1_N-6×His_* and pHKAOXm-*ADH1_N-6×His_* were also constructed through the digestion of restriction enzymes and T4 ligation, with the restriction enzyme sites at both ends of *ADH1_N-6×His_* for *Eco*RI and *Not*I. The plasmids pHKADH3-*ADH1_N-6×His_* were constructed through the digestion of restriction enzymes and seamless cloning. The P*_ADH3_* segments were amplified based on the genome of *P. pastoris* GS115, and the linearized plasmid pHKA-*ADH1_N-6×His_*, without the promoter, was obtained after the digestion by restriction enzymes *Eco*RI and *Bgl*II. Then, plasmids pHKADH3-*ADH1_N-6×His_* were obtained through seamlessly cloning the P*_ADH3_* segments into the linearized plasmid. Multicopy plasmids were also constructed through the digestion of restriction enzymes and T4 ligation. Restriction enzymes *Bgl*II and *Bam*HI were used to digest the single-copy plasmids to obtain the expression cassettes, which were inserted into the single-copy plasmids predigested with *Bgl*II to obtain the two-copy plasmids. Then, the single expression cassettes were inserted into the two-copy plasmids using similar approaches to obtain the three-copy plasmids. The detailed scheme of the construction of plasmids is provided in the Supplementary File “Construction of plasmids”. The *ADH1* gene of 1047 bp in length was PCR-amplified using the primers ADH1-N-His-EcoRI-F and ADH1-NotI-R (Supplementary Figure S1a). To determine the positive ligations of plasmids containing single or multicopy target genes with the promoters P*_AOXm_* or P*_AOX1_*, two restriction enzymes (i.e., *Bgl*II and *Xho*I) were used to digest these plasmids (Supplementary Figure S1b).

### 3.2. Expression of ADH1 in Pichia pastoris GS115

**Figure 2:**
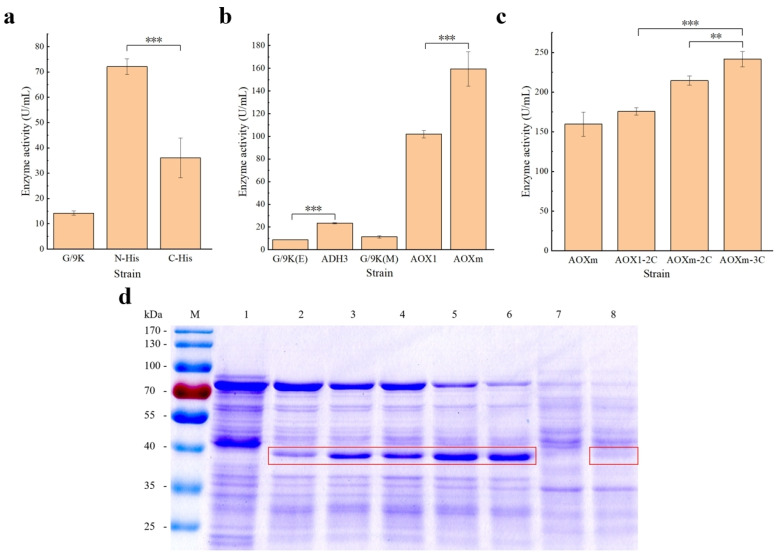
Enzymatic activity (**a**–**c**) and SDS-PAGE analysis (**d**) of different strains of *Pichia pastoris*, including the control strain GS115/pPIC9K (G/9K) and the recombinant strains GS115/pPIC9K-*ADH1_N-6×His_* (N-His) and GS115/pPIC9K-*ADH1_C-6×His_* (C-His) (**a**), as well as other strains represented by promoter name of the expression cassette (**b**) and the copy number of target gene (**c**). The strains G/9K(E) and ADH3 are cultured with ethanol as the carbon source, and G/9K(M) and other strains are cultured with methanol as the carbon source. Lane M represents the protein marker. Lanes 1 to 6 correspond to the methanol-induced strains GS115/pPIC9K, GS115/pHKA-*ADH1_N-6×His_*, GS115/pHKA-*ADH1_N-6×His_*-2Copies, GS115/pHKAOXm-*ADH1_N-6×His_*, GS115/pHKAOXm-*ADH1_N-6×His_*-2Copies, and GS115/pHKAOXm-*ADH1_N-6×His_*-3Copies, respectively. Lanes 7 and 8 correspond to strains GS115/pPIC9K and GS115/pHKADH3-*ADH1_N-6×His_*, respectively, cultured with ethanol as the carbon source. The bands of ADH1 are circled in red squares. Each experimental measurement of enzymatic activity is repeated three times and expressed as the mean ± standard deviation. The statistical significance is determined by Student’s *t* test based on *p* < 0.05 (**) and *p* < 0.01 (***), respectively.

The plasmids constructed above (Table 2) were transformed into the competent cells of *P. pastoris* GS115. Then, the expression of ADH1 was performed using positive transformants. The crude enzyme solutions were obtained after cell disruption with their enzymatic activities measured. The results showed that the enzymatic activities of strains GS115/pPIC9K-*ADH1_N-6×His_* and GS115/pPIC9K-*ADH1_C-6×His_* reached 72.08 ± 3.12 U/mL and 36.04 ± 7.80 U/mL, respectively (Figure 2a). The control strain GS115/pPIC9K was detected with a lower level of enzymatic activity at 14.24 ± 0.83 U/mL, which was probably due to the expression of intracellular enzymes with the same function as that of ADH1 in *P. pastoris*. Due to the higher enzymatic activity of strain GS115/pPIC9K-*ADH1_N-6×His_* than that of strain GS115/pPIC9K-*ADH1_C-6×His_*, the target gene *ADH1_N-6×His_* was selected in the further constructions of plasmids.

**Figure 3:**
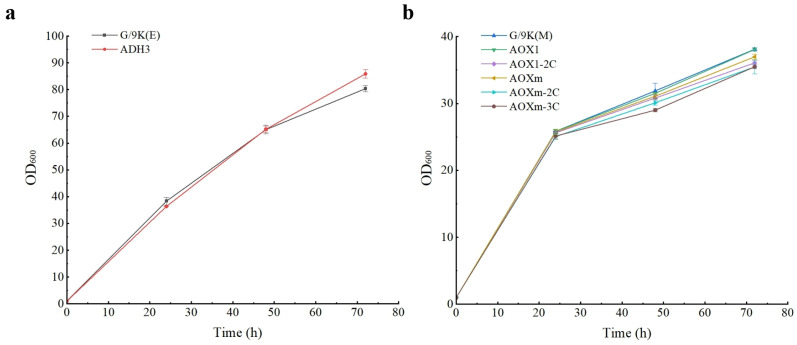
Growth curves of strains of *Pichia pastoris* with ethanol (**a**) or methanol (**b**) as the carbon sources. G/9K, the control strain GS115/pPIC9K; the remaining strains are represented by the promoter names and copy number of target gene of the expression cassette.

During the induced stage, the methanol-induced strains with P*_AOX1_* and P*_AOXm_* were supplemented with 1% methanol daily, while the ethanol-induced strains with P*_ADH3_* were supplemented with 1% ethanol daily. The control strain GS115/pPIC9K was cultured with methanol or ethanol, respectively. In 72 h cultivation, the biomass (evaluated by OD_600_) of all the strains was measured every 24 h. Different strains with the same carbon source were revealed to have similar cellular growth (Figure 3a,b), suggesting that different promoters and the three-copy gene dosage could not significantly increase the cell burden. However, the strains grown in the medium with ethanol as the carbon source were revealed to have faster growth than those using methanol as the carbon source.

The results of the enzymatic activities of all the strains showed that the enzymatic activity of strain GS115/pHKAOXm-*ADH1_N-6×His_* reached 159.48 ± 15.05 U/mL, which was higher than those of strain GS115/pHKA-*ADH1_N-6×His_* (101.81 ± 3.12 U/mL) and GS115/pHKADH3-*ADH1_N-6×His_* (23.25 ± 0.54 U/mL), respectively (Figure 2b; Supplementary Table S3). These results indicated that among these three promoters, P*_AOXm_* was the most optimal for the expression of ADH1. Therefore, the effect of increasing the copy number of P*_AOXm_*-*ADH1_N-6×His_* expression cassette on the expression of ADH1 was further investigated, showing that this approach improved the enzymatic activity of ADH1 (Figure 2c). Specifically, the enzymatic activity of strain GS115/pHKAOXm-*ADH1_N-6×His_*-3Copies reached 241.47 ± 9.49 U/mL, which was 137.18% higher than that of the original strain, i.e., GS115/pHKA-*ADH1_N-6×His_*.

The intracellular proteins extracted from the different strains were diluted four times prior to the SDS-PAGE analysis (Figure 2d). The target band of ADH1 (38 kDa) was confirmed, which was close to 40 kDa of the standard marker. The width and brightness of the target bands illustrated the enzymatic activity of ADH1 extracted in each strain. The two brightest bands were observed in strains GS115/pHKAOXm-*ADH1_N-6×His_*-2Copies and GS115/pHKAOXm-*ADH1_N-6×His_*-3Copies. The target band from strain GS115/pHKADH3-*ADH1_N-6×His_* was weak, indicating its low enzymatic activity.

### 3.3. Purification of ADH1

**Figure 4:**
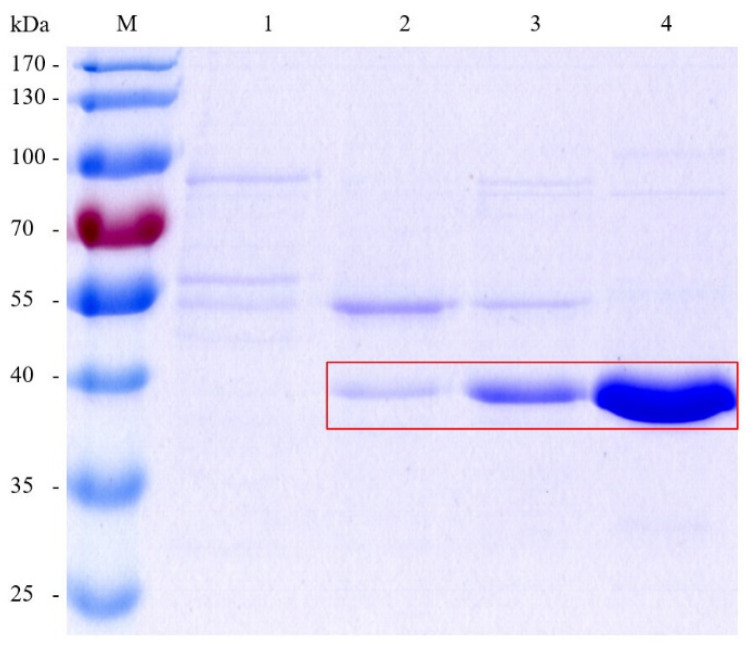
SDS-PAGE analysis of purified ADH1. Lane M: protein marker; lanes 1–4: 5%, 10%, 20%, and 50% buffer B gradient, respectively. The bands of ADH1 are circled in a red square.

A large quantity of intracellular proteins was extracted and purified. Specifically, eluents presented at 5%, 10%, 20%, and 50% buffer B gradients with protein elution peaks were collected for SDS-PAGE analysis (Figure 4). The results showed that ADH1 was eluted under 50% buffer B gradient with 150 mM imidazole and a few other proteins in the eluent. Then, the enzymatic activity of the eluent at 50% gradient was determined (221.65 ± 16.22 U/mL) with the specific enzymatic activity of 605.44 ± 44.30 U/mg.

### 3.4. Reduced Content of Acetaldehyde in Wine by ADH1

**Figure 5:**
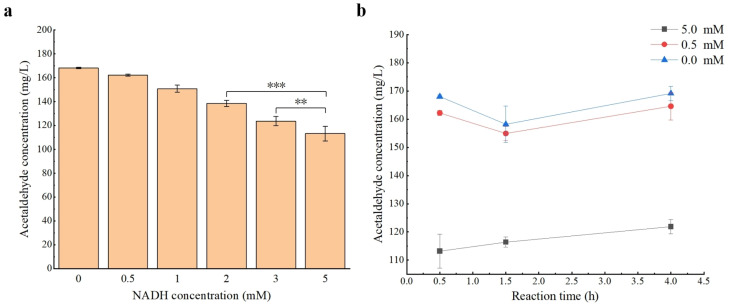
Variations in the content of acetaldehyde in wine by ADH1. (**a**) Concentration of acetaldehyde in reaction systems at 0.5 h. (**b**) Concentration of acetaldehyde in wine samples with the addition of ADH1 and NADH of different concentrations over time. Each measurement of the content of acetaldehyde is repeated three times and expressed as the mean ± standard deviation. The statistical significance is determined by Student’s *t* test based on *p* < 0.05 (**) and *p* < 0.01 (***), respectively.

The wine samples obtained from Domaine Durieu were used to evaluate the effect of ADH1 on the reduction of the acetaldehyde content in wine. Prior to the treatment of ADH1, the original concentration of acetaldehyde in the wine sample was 190.54 ± 10.69 mg/L. With the addition of NADH (i.e., the cofactor) in the conversion reaction of acetaldehyde to ethanol, the concentration of acetaldehyde in the wine samples was determined in 30 min. The results indicated that the concentration of acetaldehyde in the control group was 179.23 ± 5.51 mg/L, which was comparable to the initial concentration due to the effect of dilution, showing that the content of acetaldehyde was largely not reduced with only NADH added without the enzyme. In the experimental groups (Figure 5a), the concentration of acetaldehyde was revealed with a decreasing pattern with the increase in NADH concentration. The concentration of acetaldehyde was reduced to 113.17 ± 6.08 mg/L when the NADH concentration reached 5 mM, which was a decrease of 32.65% compared with that of the reaction system without the addition of NADH. The concentrations of acetaldehyde in the wine samples added with both ADH1 and NADH of different concentrations were tested over time (Figure 5b). The results revealed that the reaction with 5 mM NADH was completed within 0.5 h, and then the concentration of acetaldehyde tended to stabilize. The concentrations of acetaldehyde in the reaction system with 0 or 0.5 mM NADH reached the lowest levels in 1.5 h, suggesting that the reaction was completed between 0.5 and 4 h. These results indicated that ADH1 could reduce the content of acetaldehyde in wine, showing an increasing pattern in the amount of acetaldehyde reduced as the NADH concentration was increased. In addition, the application of ADH1 to reduce the acetaldehyde content of other alcoholic beverages, i.e., baijiu, has also been performed. The results also showed that the acetaldehyde content of baijiu was decreased with the catalysis of ADH1 (Figure S2), thus widening the application spectrum of ADH1.

**Figure 6:**
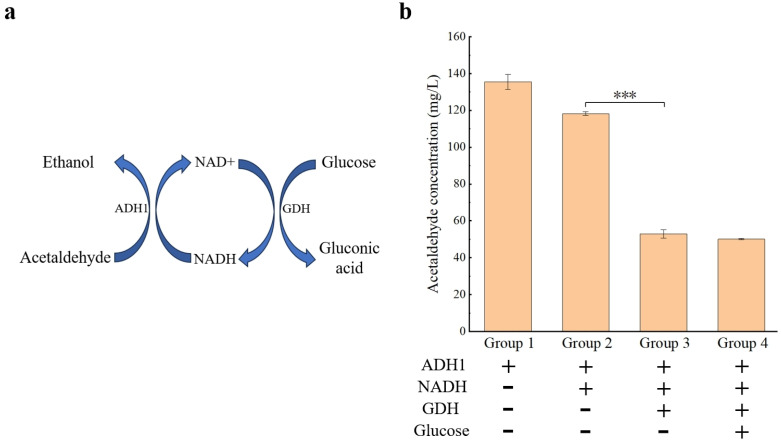
Diagram of cofactor regeneration (**a**) and the reduction in the content of acetaldehyde in four groups of wine samples by both ADH1 and GDH (**b**). Symbols “+” and “−” represent the presence and absence of the corresponding substances, respectively. Each measurement of the content of acetaldehyde is repeated three times and expressed as the mean ± standard deviation. The statistical significance is determined by Student’s *t* test based on *p* < 0.01 (***), respectively.

The content of acetaldehyde and the addition of NADH in wine were further reduced by both ADH1 and GDH, which catalyzed the conversion of glucose to gluconic acid and regenerated the cofactor NADH (Figure 6a). The results showed that with the concentration of NADH in groups 2 to 4 set to 0.5 mM, the concentration of acetaldehyde in wine was reduced to 52.89 ± 2.20 mg/L under the combined treatment of both ADH1 and GDH (Figure 6b, group 3), which was 65.34 mg/L lower than that of the reaction system with only ADH1 in group 2. The concentration of acetaldehyde was reduced to 50.08 ± 0.40 mg/L with the addition of glucose (group 4), which was close to that of group 3, suggesting that the content of glucose in the wine sample was sufficient to decrease the content of acetaldehyde. These results indicated that the effect of ADH1 on reducing the content of acetaldehyde in wine could be further synergistically improved with the addition of GDH.

## 4. Discussion

Nickel column affinity chromatography is a commonly used method for protein purification that requires the fusion expression of the target protein and His-tag [32]. However, studies have shown that His-tag could decrease the enzymatic activity of proteins [33,34]. Therefore, we comparatively evaluated the effect of the addition of His-tag at the N-terminal or C-terminal on the enzymatic activity of ADH1. The results showed that the strains with N-His-tag had higher enzymatic activity (Figure 2a), indicating that the addition of His-tag to the N terminal of ADH1 was revealed to have a lower effect on the enzymatic activity. For example, previous studies showed that the catalytic efficiency of 3-hydroxybutyrate dehydrogenase with N-His-tag was approximately 1200-fold higher than that with C-His-tag [35], while the addition of C-His-tag resulted in an eight-fold reduction in the enzymatic activity of YedY [36]. Since the effect of His-tag on enzymatic activity is related to the structure of the enzyme [35,37,38], it is necessary to determine the appropriate location on the enzyme to add His-tag.

Optimizing the promoter of the expression cassette is a commonly used method for improving the expression level of recombinant proteins in *P. pastoris* [22]. Promoters are usually selected from either natural resources or available modified promoters. For example, except for P*_AOX1_* and P*_ADH3_*, numerous types of natural promoters, i.e., methanol-induced P*_DAS1_* [39], P*_DAS2_* [39], and P*_FLD1_* [40], rhamnose-induced P*_LAR3_* [41], and constitutive promoters P*_TEF1_* [42] and P*_GCW14_* [43], have been reported to promote the strong expression of recombinant proteins. Furthermore, in addition to the modified promoter P*_AOXm_*, modifications are also made for P*_GAP_* [44] and P*_CAT1_* [45], among others, to improve the expression yield. In this study, we selected three promoters, i.e., P*_AOX1_*, P*_AOXm_* [25], and P*_ADH3_* [26], to induce the expression of ADH1. In particular, the expression level of promoter P*_AOXm_* was about 60% higher than that of P*_AOX1_* for the expression of green fluorescent protein (GFP) [25], in accordance with our results, showing an increase in the expression of ADH1 of 56.64%. Furthermore, promoter P*_ADH3_* was revealed to have a similar expression level of xylanase to that of P*_GAP_* and about 50% of the expression level of P*_AOX1_* [26]. However, in our study, the expression level of ADH1 by promoter P*_ADH3_* was only 22.86% of that of P*_AOX1_*. These results indicated that the expressions of different recombinant proteins by different promoters were different, probably relating to the amino acid sequence of recombinant proteins.

To increase the copy number of target genes is also one of the commonly applied methods to increase the expression of target proteins [22]. Multicopy strains can be constructed using either in vitro or in vivo methods. On the one hand, in the in vitro method, a vector is constructed with multiple copies of the expression cassette and then transformed into the host strain [24]. The vectors can be transformed several times by recycling the selectable marker to construct the multicopy strains [46]. On the other hand, the in vivo method is useful to screen out spontaneous multicopy strains by increasing the concentration of antibiotics. Both geneticin [47] and hygromycin [48] are frequently used for in vivo multicopy screening. In our study, multicopy strains were constructed using the in vitro method, although plasmids with more than three copies of target genes were difficult to construct. Therefore, it was recommended that the combined strategy of both in vivo and in vitro methods could be used to construct multicopy (more than three copies) strains based on the three-copy plasmids to further improve the expression level of the target genes.

Although acetaldehyde is an important flavor substance commonly found in wines [1], it exhibits adverse effects on human health. Therefore, it is important and necessary to regulate the high concentration of acetaldehyde in wines [15]. To date, the main strategies to control the content of acetaldehyde in wines are to optimize the fermentation conditions during the brewing process, such as temperature [12], ventilation [49], and the introduction of lactic acid bacteria [50]. Studies have shown that appropriately increased temperature during fermentation could promote the fermentation of microorganisms, thus promoting the formation of acetaldehyde [12], while high temperatures could promote the volatilization of acetaldehyde [51], suggesting that the content of acetaldehyde in wines could be regulated by adjusting the temperature during fermentation. Furthermore, the appropriate increase in ventilation during fermentation could accelerate the oxidation of wine and increase the content of acetaldehyde [52]. Moreover, previous studies showed that the content of acetaldehyde during winemaking could be significantly reduced by introducing two commercial starter cultures of *Oenococcus oeni* [15]. However, the application of this method affected not only the content of acetaldehyde but also the contents of other substances in wines [12,49], ultimately making an impact on wine flavor. Therefore, in this study, we explored a novel strategy to reduce the content of acetaldehyde in wines after fermentation based on the enzymatic property of ADH1, i.e., catalyzing the conversion reaction of acetaldehyde to ethanol [17], which is a major chemical component in wines, thereby exhibiting significant potential to be applied in the wine industry to reduce the content of acetaldehyde in wines. One of the main advantages of this method is that it is conducive to the precise control of the content of acetaldehyde with less influence on other substances in wines.

Since the conversion between acetaldehyde and ethanol in the presence of cofactors is a reversible reaction [17] and the content of ethanol in wines is generally much higher than that of acetaldehyde [53], a large quantity of NADH is needed for the reaction to promote ethanol synthesis. Indeed, the conversion rate is closely related to the content of NADH in the reaction system [54]. Our study showed that the concentration of acetaldehyde was no longer decreased when the reaction reached an equilibrium state (Figure 5b). However, the economic benefits of reducing the content of acetaldehyde in wines may not be sufficient to compensate for the cost of NADH application due to its high price [55]. Therefore, the recycling of both NADH and NAD^+^ should be considered during the process of reducing the content of acetaldehyde using ADH1, which could be performed by coupling with another enzyme (e.g., GDH) and converting the cofactor NAD^+^ into NADH [55]. Our results showed that with the addition of GDH and NADH at low concentrations of 0.5 mM, ADH1 was revealed to have an optimal effect on reducing the content of acetaldehyde (Figure 6b). Furthermore, gluconic acid, which is the catalytic product of GDH, is also a common substance found in wines [56]. Therefore, both GDH and ADH1 could be synergistically applied to appropriately reduce the content of glucose in wines, ultimately reducing the content of acetaldehyde in wines. Finally, further studies could consider immobilizing these two enzymes and cofactors to recover the enzymes and cofactors [57] and to decrease the addition of NADH, providing an effective strategy for reducing the content of acetaldehyde in wines through enzymatic catalysis.

Based on the fact that ADH1 could catalyze the reduction of acetaldehyde to ethanol [17], the content of acetaldehyde in wine could be reduced by the exogenous addition of ADH1. In our study, the role of NADH in the reaction was verified, i.e., the acetaldehyde concentration was decreased as the content of NADH was increased (Figure 5a), and the acetaldehyde content in wine could be further reduced through the establishment of a cofactor cycle (Figure 6b). Indeed, previous studies adopted the strategy of metabolic engineering to reduce acetaldehyde synthesis during fermentation by overexpressing ADH1 [58] or other enzymes involved in NADH synthesis in yeast [59,60]. For example, in beer fermentation, the overexpression of ADH1 could reduce the acetaldehyde content of beer from 9.51 ± 0.09 mg/L to 6.93 ± 0.09 mg/L [58], a decrease of 27.13%. Through the overexpression of the citrate synthetase (CIT1), the increase in the intracellular NADH/NAD+ ratio reduced the acetaldehyde content of beer from 13.26 ± 0.36 mg/L to 7.28 ± 0.14 mg/L [61], which was a decrease of 45.10%. Therefore, the overexpression of ADH1 and other enzymes involved in the pathways of NADH synthesis in strains used in winemaking could be an alternative strategy. This strategy involves reducing the synthesis of acetaldehyde in the fermentation process and has the economic advantage because it does not require exogenous NADH [58]. However, the method of exogenous addition of ADH1 adopted in our study has other advantages in terms of conversion rate. In particular, by constructing a cofactor cycle, the acetaldehyde content was reduced from 135.53 ± 4.08 mg/L to 52.89 ± 2.20 mg/L (Figure 6b), a decrease of 60.98%, which was higher than the conversion rate of the overexpression strategy. The conversion rate can also be necessarily controlled by adjusting the concentration of NADH or the reaction time (Figure 5), because acetaldehyde is an important flavor substance in wine, and its concentration should be maintained in a proper range [1,5]. However, it is difficult to accurately control the acetaldehyde content through the overexpression strategy. Furthermore, because the method of exogenous addition is not associated with the regulation of alcohol fermentation, it could be used to reduce the acetaldehyde content of wine after fermentation, thus expanding the scope of its application.

## 5. Conclusions

In this study, alcohol dehydrogenase I (ADH1) from *S. cerevisiae* was successfully expressed in *P. pastoris*, showing the higher enzymatic activities of strains with the expression of *ADH1_N-6×His_* compared with those of *ADH1_C-6×His_*. These results indicated that it was more appropriate to add the 6×His-tag to the N terminal of ADH1 rather than the C terminal. Furthermore, replacing the promoter with a stronger promoter, i.e., P*_AOXm_*, and increasing the copy number of the target genes resulted in an increase of 137.18% in the enzymatic activity compared with the original strain. Based on the nickel column affinity chromatography purification, ADH1 was eluted under the buffer of imidazole at a concentration of 150 mM, while the specific enzymatic activity of the resulting solution reached 605.44 ± 44.30 U/mg. Moreover, ADH1 could significantly decrease the content of acetaldehyde in wines with the addition of both cofactors NADH and glucose dehydrogenase (GDH), showing the synergistic effect of the enzyme catalytic method on reducing the content of acetaldehyde in wines. The method of the exogenous addition of ADH1 to reduce the acetaldehyde in wine provides a new approach for reducing the content of acetaldehyde in wine, which could precisely regulate the acetaldehyde content in wine after fermentation.

## Figures and Tables

**Table 1:** Common methods for regulating acetaldehyde content in wine.

Condition	Effect	Efficiency	Reference
Temperature	With the fermentation temperature adjusted from 16 to 24 °C, the acetaldehyde content is decreased from 19 to 13 mg/L	31.58%	[12]
Ventilation	The acetaldehyde content is decreased from 10.92 to 4.21 mg/L in low-level oxygenation compared with high-level oxygenation	61.45%	[13]
SO_2_	The addition of 30 mg/L SO_2_ increases the acetaldehyde content from 24 to 36 mg/L	33.33%	[14]
Microorganism	By introducing *Oenococcus oeni* into the malolactic fermentation, the acetaldehyde content of white wine is reduced from 90 mg/L to almost none	Over 90%	[15]

**Table 2:** Plasmids constructed in this study.

Plasmid	Expression Cassette	Copy Number of Gene *ADH1*
pPIC9K-*ADH1_N-6×His_*	P*_AOX1_*-*ADH1_N-6×His_*-AOX1 TT	1
pPIC9K-*ADH1_C-6×His_*	P*_AOX1_*-*ADH1_C-6×His_*-AOX1 TT	1
pHKA-*ADH1_N-6×His_*	P*_AOX1_*-*ADH1_N-6×His_*-AOX1 TT	1
pHKA-*ADH1_N-6×His_*-2Copies	P*_AOX1_*-*ADH1_N-6×His_*-AOX1 TT	2
pHKAOXm-*ADH1_N-6×His_*	P*_AOXm_*-*ADH1_N-6×His_*-AOX1 TT	1
pHKAOXm-*ADH1_N-6×His_*-2Copies	P*_AOXm_*-*ADH1_N-6×His_*-AOX1 TT	2
pHKAOXm-*ADH1_N-6×His_*-3Copies	P*_AOXm_*-*ADH1_N-6×His_*-AOX1 TT	3
pHKADH3-*ADH1_N-6×His_*	P*_ADH3_*-*ADH1_N-6×His_*-AOX1 TT	1

## Data Availability

The dataset is available upon the request of the corresponding authors.

## Supplementary Materials

The following supporting information can be downloaded at: https://www.mdpi.com/article/10.3390/microorganisms12010038/s1, Figure S1: PCR verification of *ADH1* and the double-enzyme digestion of plasmids; Figure S2: Variations in the content of acetaldehyde in baijiu based on ADH1; Table S1: Primers and their sequences used in this study; Table S2: The reaction systems of the reduction of the content of acetaldehyde in four groups of wine samples by ADH1 and GDH; Table S3: Enzymatic activities of ADH1 in all strains of *Pichia pastoris*; Construction of plasmids; Amplification of *ADH1* gene and double enzyme digestion of plasmids; Reduced content of acetaldehyde in baijiu by ADH1.
