# Supplementary material for: Enhanced Expression of Alcohol Dehydrogenase I in Pichia pastoris Reduces the Content of Acetaldehyde in Wines
Source: Microorganisms. 2023 Dec 25;12(1):38. doi: 10.3390/microorganisms12010038 (PMC10820543; doi:10.3390/microorganisms12010038)
Supplement: Supplementary file 1 [file microorganisms-12-00038-s001.zip › microorganisms-2729863-supplementary.pdf]

microorganisms

MDPI

Article

Enhanced Expression of Alcohol Dehydrogenase I in *Pichia pastoris* Reduces the Content of Acetaldehyde in Wines

Kun Geng[1,2], Ying Lin[1,2], Xueyun Zheng[3], Cheng Li[4], Shuting Chen[1,2], He Ling[1,2], Jun Yang[1,2], Xiangyu Zhu[1,2] and Shuli Liang[1,2,*]

[1] School of Biology and Biological Engineering, South China University of Technology, Guangzhou 510006, China
[2] Guangdong Provincial Key Laboratory of Fermentation and Enzyme Engineering, South China University of Technology, Guangzhou 510006, China
[3] Key Laboratory of Fermentation Engineering of Ministry of Education, School of Biological Engineering and food, Hubei University of Technology, Wuhan 430068, China
[4] Department of Biology, Massachusetts Institute of Technology, Cambridge, MA 02139, USA
* Correspondence: shuli@scut.edu.cn

Supplementary File: The codon-optimized sequence of ADH1 (5′→3′)

ATGTCTATTCCAGAAACTCAAAAGGGAGTTATTTTTTACGAATCACATGGAAAGTTGGAATACAAGGATATTC
CAGTTCCTAAGCCAAAGGCTAACGAATTGTTGATTAACGTTAAGTACTCAGGAGTTTGTCATACTGATCTGCA
CGCTTGGCATGGAGATTGGCCATTGCCAGTTAAGTTGCCTTTGGTTGGAGGTCATGAAGGTGCTGGTGTTGTTG
TTGGTATGGGAGAAAACGTTAAGGGATGGAAGATTGGAGATTACGCTGGTATTAAGTGGTTGAACGGATCAT
GTATGGCTTGTGAATACTGTGAATTGGGTAACGAATCAAACTGTCCACATGCTGATTTGTCCGGTTACACTCA
TGATGGTAGCTTTCAACAATACGCTACTGCTGATGCTGTTCAAGCTGCTCATATTCCACAAGGAACTGATTTG
GCTCAAGTTGCTCCAATTTTGTGTGCTGGTATTACTGTTTACAAGGCTTTGAAGTCCGCTAACTTGATGGCTGG
ACATTGGGTTGCTATTTCAGGAGCTGCTGGAGGATTGGGCTCCTTGGCTGTTCAATACGCTAAGGCTATGGGT
TACAGAGTTTTGGGTATTGATGGAGGTGAAGGTAAGGAAGAATTGTTTAGATCCATTGGAGGTGAAGTTTTTA
TTGATTTTACTAAGGAAAAGGATATTGTTGGTGCTGTTTTGAAGGCTACTGATGGTGGTGCTCATGGAGTTATT
AACGTTTCCGTTTCAGAAGCTGCTATTGAAGCCTCCACTAGATACGTTAGAGCTAACGGAACTACTGTTTTGG
TTGGTATGCCTGCTGGTGCTAAGTGTTGTTCCGATGTTTTTAACCAAGTTGTTAAGTCCATTTCTATTGTTGGAT
CTTACGTTGGTAACAGAGCTGATACTAGAGAGGCGTTGGATTTTTTTGCTAGAGGTTTGGTTAAGTCACCAATT
AAGGTTGTTGGTTTGTCAACTTTGCCAGAAATTTACGAAAAGATGGAAAAGGGACAAATTGTTGGTAGATAC
GTTGTTGATACTTCTAAGTAA

Supplementary Table S1. Primers and their sequences used in this study. "-F" and "-R" indicate the forward and reverse primers, respectively.

Primer	Sequence (5′→3′)
ADH1-N-His-BamHI-F	CGCGGATCCATGCATCATCATCATCATCATTCTATTCCAGAAACTCAAAA GGGAG
ADH1-NotI-R	AAGGAAAAAAGCGGCCGCTTACTTAGAAGTATC
ADH1-BamHI-F	CGCGGATCCATGTCTATTCCAGAAACTCAAAAGGGAG
ADH1-C-His-NotI-R	TTCGCGGCCGCTTAATGATGATGATGATGATGCTTAGAAGTATCAACAA CG
ADH1-N-His-EcoRI-F	CCGGAATTCATGCATCATCATCATCATCATTCTATTCC
pHKA-ADH3-F	GGATTTTGGTCATGAGATCAGATCTTCTGACGGTACTAGAGGAC
pHKA-ADH3-R	GATGATGATGATGATGCATGAATTCCGTAAAGTAAATAAGATAA

Copy-F	GCGCTCTGCTGAAGCCAGTTACCTTCGGAA
Copy-R	GGCAGTACCGGCATAACCAAGCCTATGCCT

Supplementary Table S2. The reaction systems of the reduction in the contents of acetaldehyde in four groups of wine samples by ADH1 and GDH. Symbol "—" indicates no addition of the specific substances.

**Group**	**Wine sample (μL)**	**ADH1 (μL)**	**NADH (μL)**	**GDH (μL)**	**Glucose (μL)**	**ddH$_2$O (μL)**
1	1400	300	—	—	—	300
2	1400	300	100	—	—	200
3	1400	300	100	100	—	100
4	1400	300	100	100	100	—

Supplementary Table S3. Enzymatic activities of ADH1 in all strains of *Pichia pastoris*.

**Strain**	**Enzymatic activity (U/mL)**
GS115/pPIC9K (Methanol)	14.24 ± 0.83
GS115/pPIC9K (Ethanol)	8.65 ± 0.01
GS115/pPIC9K-$ADH1_{N\text{-}6\times His}$	72.08 ± 3.12
GS115/pPIC9K-$ADH1_{C\text{-}6\times His}$	36.04 ± 7.80
GS115/pHKA-$ADH1_{N\text{-}6\times His}$	101.81 ± 3.12
GS115/pHKA-$ADH1_{N\text{-}6\times His}$-2Copies	175.69 ± 4.68
GS115/pHKAOXm-$ADH1_{N\text{-}6\times His}$	159.48 ± 15.05
GS115/pHKAOXm-$ADH1_{N\text{-}6\times His}$-2Copies	214.44 ± 5.63
GS115/pHKAOXm-$ADH1_{N\text{-}6\times His}$-3Copies	241.47 ± 9.49
GS115/pHKADH3-$ADH1_{N\text{-}6\times His}$	23.25 ± 0.54

Strains GS115/pPIC9K (Ethanol) and GS115/pHKADH3-$ADH1_{N\text{-}6\times His}$ are cultured with ethanol as the carbon source; GS115/pPIC9K (Methanol) and other strains are cultured with methanol as the carbon source.

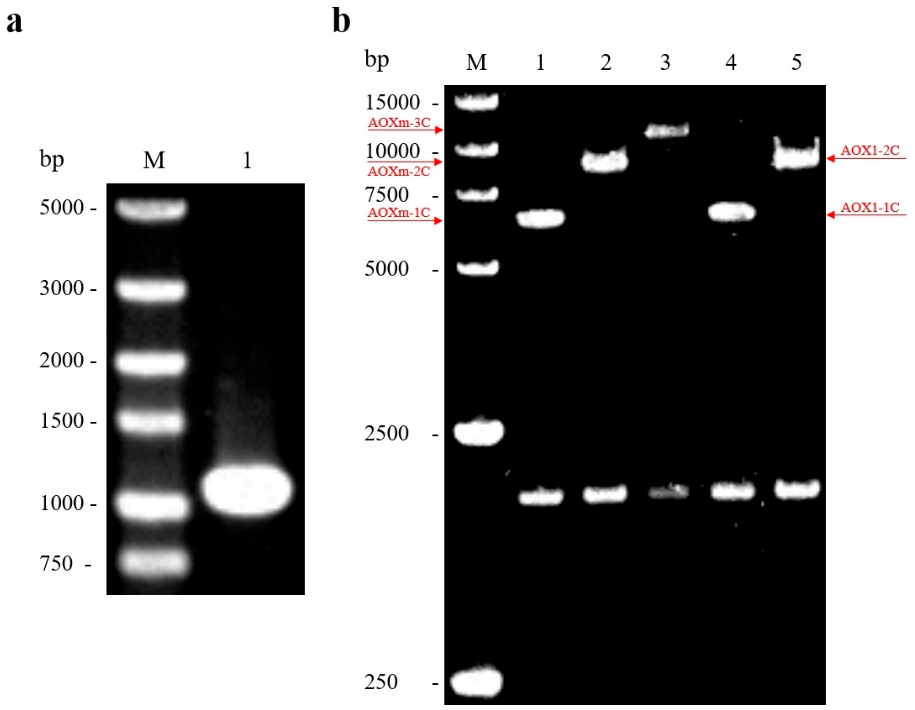

Supplementary Figure S1. PCR verification of *ADH1* and the double-enzyme digestion of plasmids. (a) PCR-amplification of *ADH1*$_{N-6\times His}$. (b) Length of the segments of the digested plasmids with multicopy target genes. Lane M: the DNA marker; lanes 1–3 represent the segments of digested plasmids pHKAOXm-*ADH1*$_{N-6\times His}$, pHKAOXm-*ADH1*$_{N-6\times His}$-2Copies, and pHKAOXm-*ADH1*$_{N-6\times His}$-3Copies, respectively; lanes 4 and 5 contain the segments of digested plasmids pHKA-*ADH1*$_{N-6\times His}$ and pHKA-*ADH1*$_{N-6\times His}$-2Copies, respectively. The longer segment following double-enzyme digestion of each plasmid is marked with a red arrow.

Construction of plasmids

Both pPIC9K-*ADH1*$_{N-6\times His}$ and pPIC9K-*ADH1*$_{C-6\times His}$ were constructed as follows: genes *ADH1*$_{N-6\times His}$ and *ADH1*$_{C-6\times His}$ were PCR-amplified using the primer pairs ADH1-N-His-BamHI-F/ADH1-NotI-R and ADH1-BamHI-F/ADH1-C-His-NotI-R, respectively. Then, these segments and the plasmid pPIC9K were digested with the restriction enzymes *Bam*HI and *Not*I. Finally, the digested segments were ligated to obtain the recombinant plasmids pPIC9K-*ADH1*$_{N-6\times His}$ and pPIC9K-*ADH1*$_{C-6\times His}$.

Promoters P$_{AOX1}$, P$_{AOXm}$, and P$_{ADH3}$ were used to induce the expression of ADH1, with P$_{AOX1}$ and P$_{AOXm}$ inserted into the plasmid pHKA. The primers ADH1-N-His-EcoRI-F and ADH1-NotI-R were used to amplify the gene *ADH1*$_{N-6\times His}$. Then, both plasmids pHKA and pHKAOXm and *ADH1*$_{N-6\times His}$ segments were digested by the restriction enzymes *Eco*RI and *Not*I, with the digested segments ligated to obtain the recombinant plasmids pHKA-*ADH1*$_{N-6\times His}$ and pHKAOXm-*ADH1*$_{N-6\times His}$. The P$_{ADH3}$ segments were amplified based on the genome of *Pichia pastoris* GS115 using primers pHKA-ADH3-F and pHKA-ADH3-R. Then, the linearized plasmid pHKA-*ADH1*$_{N-6\times His}$, without the promoter, was obtained after the digestion by restriction enzymes *Eco*RI and *Bgl*II. To summarize, the plasmid pHKADH3-*ADH1*$_{N-6\times His}$ was constructed by replacing the original promoter P$_{AOX1}$ with P$_{ADH3}$.

Multicopy plasmids were constructed as follows: restriction enzymes *Bgl*II and *Bam*HI were used to digest the plasmids pHKA-*ADH1*$_{N-6\times His}$ and pHKAOXm-*ADH1*$_{N-6\times His}$ to obtain the expression cassettes, which were inserted into the single-copy plasmids pHKA-*ADH1*$_{N-6\times His}$ and pHKAOXm-*ADH1*$_{N-6\times His}$ predigested with *Bgl*II, respectively, to obtain the two-copy plasmids pHKA-*ADH1*$_{N-6\times His}$-2Copies and pHKAOXm-*ADH1*$_{N-6\times His}$-2Copies, respectively. Finally, the three-copy

plasmid pHKAOXm-$ADH1_{N\text{-}6\times His}$-3Copies was constructed using the similar approaches to that for the construction of the two-copy plasmids. The single expression cassettes were successfully inserted into the single-copy or two-copy plasmids due to the same sticky ends generated by *Bgl*II or *Bam*HI.

Amplification of *ADH1* gene and double enzyme digestion of plasmids

The primers ADH1-N-His-EcoRI-F and ADH1-NotI-R were used to amplify the *ADH1* gene, which was 1,047 bp in length. and the resulting segments after digestion with *Eco*RI and *Not*I were confirmed (Supplementary Figure S1a). To determine the positive ligations of plasmids containing single or multicopy target genes with the promoters P_{AOXm} or P_{AOX1}, two restriction enzymes (*Bgl*II and *Xho*I) were used to digest these plasmids. The lengths of the P_{AOXm} and P_{AOX1} expression cassettes were 2,382 bp and 2,434 bp, respectively. After the digestion with *Bgl*II and *Xho*I, the fragments of the plasmid with the single expression cassette P_{AOXm}-*ADH1* were 6,509 bp and 1,985 bp, the fragments of the plasmid with a two-copy expression cassette were 8,891 bp and 1,985 bp, and the fragments of the plasmid with a three-copy expression cassette were 11,273 bp and 1,985 bp, respectively. Similarly, the fragments of the plasmid with the single expression cassette P_{AOX1}-*ADH1* were 6,561 bp and 1,985 bp, and the fragments of the plasmid with the two-copy P_{AOX1}-*ADH1* were 8,995 bp and 1,985 bp, respectively (Supplementary Figure S1b).

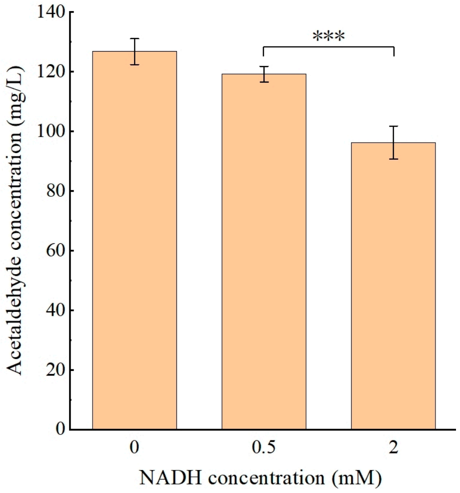

Supplementary Figure S2. Variations in the content of acetaldehyde in baijiu based on ADH1. Concentration of acetaldehyde in reaction systems is tested at 4 h. Each measurement of the content of acetaldehyde is repeated three times and expressed as the mean ± standard deviation. The statistical significance is determined by Student's *t* test based on $P < 0.01$ (***).

Reduced content of acetaldehyde in baijiu by ADH1

The baijiu samples obtained from Jiangji Winery were used to evaluate the effect of ADH1 on the reduction of the acetaldehyde content. The acetaldehyde concentrations of the samples with the addition of 0.5 mM and 2 mM NADH were decreased after 4 h of ADH1 catalysis, with the higher level of effect observed under the treatment of 2 mM NADH.
